# Microfluidic Systems for Isolation of Spermatozoa from Testicular Specimens of Non-Obstructive Azoospermic Men: Does/Can It Improve Sperm Yield?

**DOI:** 10.3390/jcm10163667

**Published:** 2021-08-19

**Authors:** Gary D. Smith, Clementina Cantatore, Dana A. Ohl

**Affiliations:** 1 Reproductive Sciences Program, Departments of Obstetrics/Gynecology, Physiology, and Urology, University of Michigan, Ann Arbor, MI 48103, USA; 2 Reproductive and IVF Unit, Department of Maternal and Child Health, Asi Bari, 70014 Conversano (BA), Italy; clemecant@yahoo.it; 3 Department of Urology, University of Michigan, Ann Arbor, MI 48103, USA; daohl@med.umich.edu

**Keywords:** non-obstructive azoospermia, testicular spermatozoa, processing, microfluidics, new technologies

## Abstract

Intracytoplasmic sperm injection (ICSI) has allowed reproduction options through assisted reproductive technologies (ARTs) for men with no spermatozoa within the ejaculate (azoospermia). In men with non-obstructive azoospermia (NOA), the options for spermatozoa retrieval are testicular sperm extraction (TESE), testicular sperm aspiration (TESA), or micro-surgical sperm extraction (microTESE). At the initial time of spermatozoa removal from the testis, spermatozoa are immobile. Independent of the means of spermatozoa retrieval, the subsequent steps of removing spermatozoa from seminiferous tubules, determining spermatozoa viability, identifying enough spermatozoa for oocyte injections, and isolating viable spermatozoa for injection are currently performed manually by laboratory microscopic dissection and collection. These laboratory techniques are highly labor-intensive, with yield unknown, have an unpredictable efficiency and/or success rate, and are subject to inter-laboratory personnel and intra-laboratory variability. Here, we consider the potential utility, benefits, and shortcomings of developing technologies such as motility induction/stimulants, microfluidics, dielectrophoresis, and cell sorting as andrological laboratory add-ons to reduce the technical burdens and variabilities in viable spermatozoa isolation from testicular samples in men with NOA.

## 1. NOA Background

Clinical infertility is a disease of the reproductive system defined by the failure to achieve a clinical pregnancy after 12 months or more of regularly unprotected sexual intercourse [1]. The worldwide prevalence of clinical infertility is approximately 9%, with 56% of couples seeking medical interventions [2]. Male factor infertility describes couples in whom the inability to conceive is associated with compromised reproductive function in the male partner. Broadly, this can be due to (1) compromised semen parameters involving semen volume, sperm numbers, motility, morphology, or viability; (2) abnormal sperm function; or (3) normal semen/sperm parameters, yet conditions that prevent sperm deposition in the vagina during intercourse involving male reproductive tract obstructions and/or ejaculatory dysfunction [3]. Males are solely responsible for approximately 20–30% of these clinical infertility cases and contribute to approximately 50% of cases overall (male factor and female factor). The absence of sperm in an ejaculate is termed azoospermia and occurs in less than 1% of the general male population and an estimated 10% of men with infertility. Azoospemia may be caused by an obstruction of the male reproductive tract, which is termed obstructive azoospermia (OA) and makes up approximately 40% of azoospermic cases [4]. Additionally, azoospermia may be a result of inadequate spermatogenesis in the seminiferous tubules of the testis, which is termed non-obstructive azoospermia (NOA). The introduction of intracytoplasmic sperm injection (ICSI; injection of a single sperm into a single oocyte) revolutionized the treatment of male factor infertility, OA, and NOA [5,6,7]. NOA is considered the most severe and difficult form of azoospermia to treat with assisted reproductive technologies (ARTs) for at least three primary reasons: (1) the method of gamete retrieval; (2) the variable and unpredictable degree of compromised spermatogenesis and success of spermatozoa retrieval/isolation; (3) the initial non-motile nature of retrieved testicular sperm. In this review, we will not address the pros and cons of gamete retrieval methods (as this is specifically addressed in other manuscripts within this series). However, in men with NOA, the following questions arise: (1) Are there focal sites of spermatogenesis within the testes available for spermatozoa isolation? (2) What is the best method to access these focal sites of spermatogenesis? In early studies, testicular sperm extraction (TESE) was used, whereby a single-site testicular biopsy was performed in attempting spermatozoa isolation [8,9,10]. A retrospective review of first-TESE in NOA from 1994 to 2009 (714 cycles) demonstrated 41% success of spermatozoa retrieval [11]. A modified approach to TESE is testicular sperm aspiration (TESA), which involves the placement of a needle (often a butterfly needle) with negative pressure into the testis, aspiration of fluid and tissue, and movement into multiple regions of the testis without removal from the testes, to “sample” fluid and tissue from numerous focal areas [12]. Subsequent studies using TESA reported variance in spermatozoa retrieval success ranging from 59% [13] to 54% [14]. In contrast to TESE and TESA, microTESE is another form of spermatozoa isolation in NOA. This procedure involves a urologist/surgeon bisecting the testis and using surgical microscopy and 15–20× magnification to identify and isolate dilated/plump seminiferous tubules. Though this surgical procedure is considered more invasive than TESE and TESA, it is a regionally selective biopsy of visualized and isolated seminiferous tubules—resulting in less tissue removal and the ability for spermatozoa identification from isolated tubules to be confirmed by an andrologist in the surgical suite. MicroTESE-isolated seminiferous tubules are subsequently placed into 37 °C processing media and transported to the andrology laboratory, where they are manually dissected under microscope observation. Ramasamy and colleagues [15] demonstrated that the success of spermatozoa retrieval by microTESE diminished with greater operative time, yet overall was successful in 52% of cases. The success of spermatozoa isolation from microTESE-isolated seminiferous tubules was shown to be highest in cases of dilated/plump tubule selective biopsy (90%) versus non-dilated tubule removal (7%) [16].

## 2. Current Laboratory Techniques for Spermatozoa Isolation from NOA Testicular Samples

**Figure 1:**
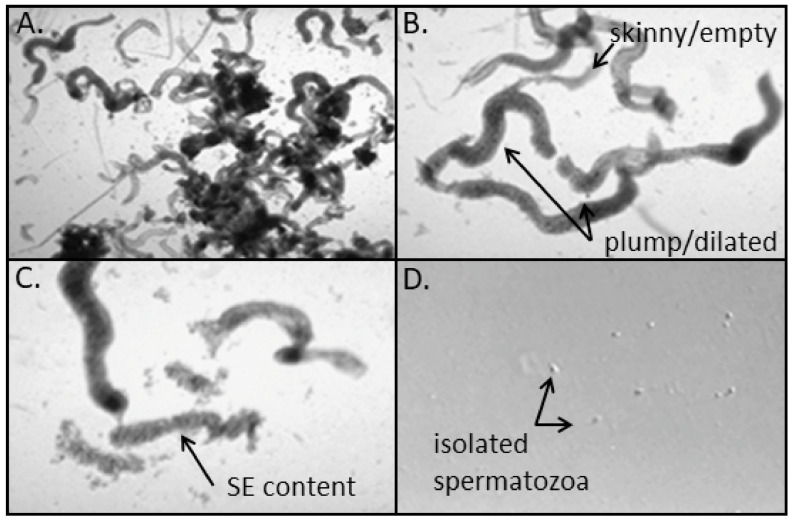
Composite micrographs of laboratory manual processing of testicular aspirate for spermatozoa isolation. (**A**) Minced seminiferous tubules (ST) in processing media. (**B**) Isolated truncated ST with indication of plump/dilated ST with presumed active spermatogenesis and skinny/empty ST (also transparent) with presumed absence of spermatogenesis. (**C**) Seminiferous tubules with one ST processed with tuberculin syringe and needles to squeeze out seminiferous epithelium (SE). (**D**) Following pulled glass pipet dispersion of SE into single cells, the isolation of non-motile testicular spermatozoa. Magnifications: (**A**)—100×, (**B**,**C**)—200×, (**D**)—400×.

Independent of the process used to collect testicular tissue/seminiferous tubules (TESE, TESA, or microTESE), the first steps in the andrology laboratory are to isolate and evaluate seminiferous tubules for the presence of active spermatogenesis (dilated/plump) or lack of spermatogenesis (not dilated/skinny/transparent; Figure 1A). Compared to TESA samples, the amount of testicular somatic or connective tissue is higher in TESE samples, which can make this process of manual seminiferous tubule isolation more difficult. TESA samples tend to yield individualized seminiferous tubules that resemble “unraveled yarn” in the collection media/tube. Within the laboratory, both TESE and TESA samples can require mincing in 37 °C processing media (simple HEPES-buffered media such as human tubule fluid (HTF)-HEPES + protein (human serum albumin (HSA)) to: (1) release the seminiferous tubules from connective tissue; (2) reduce the size of individualized seminiferous tubules; (3) produce clean-cut edges to seminiferous tubules that will facilitate collection of tubule contents. Mincing can be performed on a dissecting microscope in a drop of processing media, with tissue placed on a Petri dish with tweezers and a scalpel. This manual mincing method is useful for seminiferous tubule isolation from TESE samples. One can use a similar manual method for TESA samples or use tuberculin syringe/26–27-gauge needles to cut tubules into manageable sizes. Due to the surgical and selective nature of seminiferous tubule isolation in micro-TESE, the tubules are already isolated in a truncated and pure state and usually do not require laboratory tubule isolation and mincing.

Once the individualized dilated/plump tubules are isolated and truncated, they can be moved into a clean drop of 37 °C processing media (Figure 1B) for further manual processing. At this point, under microscopic observation, a pair of tuberculin syringe/26–27-gauge needles can be used to squeeze the seminiferous tubule contents out of each short dilated/plump tubule segment (Figure 1C). This results in seminiferous tubule content that can be aspirated easily into a small-bore (~15–20 µm inner diameter) flame-pulled glass pipet. This allows one to expel the seminiferous tubule contents into a separate fresh drop of 37 °C processing media as a single-cell suspension, which will contain germ cells of varying degrees of development, supportive cells, and—hopefully—mature spermatozoa (Figure 1D). These testicular-isolated spermatozoa will likely be non-motile; this is especially observed in samples from NOA men [17].

The culture time and conditions have been evaluated for both OA and NOA spermatozoa samples, demonstrating that 24–48 h culture of testicular spermatozoa in complex media (Ham’s F10 + albumin) can benefit spermatozoa maturation and motility induction [18,19,20]. These maturation conditions can be tested on individuals having testicular spermatozoa retrieval in a diagnostic manner, prior to a therapeutic procedure coordinated with egg retrieval. These “diagnostic” testicular spermatozoa retrievals, maturation/motility initiation, and cryopreservation of isolated “rare” spermatozoa [21,22,23] can be quite successful and have been nicely reviewed and critiqued [24].

The above-described laboratory procedure of spermatozoa isolation from testicular samples relies primarily on manual, mechanical, microscopic processing and can be quite labor- and time-intensive. There are numerous limitations to the current mechanical method of spermatozoa isolation from testicular samples that need to be addressed. The success and efficiency of spermatozoa isolation are influenced by human experience, examiner fatigue, and the slight procedural variations used to yield single-cell suspensions and visualize individual spermatozoa. These laboratory microscopic mechanical procedures can take 2–12 h, depending on testicular sample purity and volume, number of spermatozoa, volumes of media used for procedures, and laboratory personnel experience. As time spent searching for spermatozoa increases, the success of spermatozoa isolation decreases, which can impact subsequent pregnancy rates [15]. Cases with spermatozoa isolation taking < 2 h had significantly higher pregnancy rates (89%) compared to cases taking > 4 h (37%). There have been numerous reports of using enzymatic treatment of testicular samples to aid the recovery of testicular spermatozoa [25,26,27]; however, its advantages are debated. These enzymatic techniques usually use collagenase type IA or IV to digest the collagen within the basement membrane and extracellular matrix within seminiferous tubules. However, these collagenases have been demonstrated to digest cell surface proteins [28] that may have influence on downstream sperm function in fertilization, pronuclear formation, and embryo development. Additionally, enzymatic methods incorporate centrifugation, which, as discussed below, can have a detrimental impact on spermatozoa DNA integrity. Before discussion of the potential future of microfluidics for spermatozoa isolation from testicular samples of men with NOA, we need to acknowledge that achieving a level of single-cell suspension (as discussed above) will still be required; thus, a significant amount of mechanical and manual processing is still required.

## 3. Microfluidics and Potential Use in Spermatozoa Isolation from NOA Testicular Samples

Microfluidics is defined as a multidisciplinary field of study and design whereby fluid behaviors are accurately controlled and manipulated with small-scale geometric constraints that yield dominance of surface forces over volumetric forces. While past procedures in the ART laboratory have been successful, they have been more macroscale approaches to microscale cellular biological events [29]. Integration of microfluidics into the ART laboratory has at least four foreseeable advantages: (1) allowing precisely controlled fluidic gamete/embryo manipulations; (2) providing biomimetic environments for culture; (3) facilitating microscale genetic and molecular bioassays; (4) enabling miniaturization and automation. The basic utility and advantages of individual microfluidic devices for isolation of motile spermatozoa have been studied and reported over the last two decades. These can generally be categorized as microfluidic means of motile sperm isolation by three similar but slightly discrete biophysical means. 

First, motile sperm can be enriched by using a microfluidic-generated laminar flow and sperm motility-enabled crossing of the meniscus or interstream line formed by the laminar flow [30,31]. These devices allow a separation of motile spermatozoa from seminal plasma, non-motile sperm, dead cells, and debris without centrifugation or resulting potential lethal and sublethal spermatozoa damage. The technical parameters of the device were designed to optimize the isolation of motile human spermatozoa with the inflow channel (semen; 100 μm × 50 μm; width × depth), inflow channel (media; 300 μm × 50 μm), common mid-channel (laminar flow; 500 μm × 50 μm × 100 mm length). Centrifugation can negatively influence sperm motility [32], mitochondrial function [33], intact acrosomal status [33], and DNA integrity [34]. Using the microfluidic laminar flow and inertia spermatozoa isolation, it was demonstrated that isolated motile spermatozoa had significantly less DNA damage compared to processing sperm with centrifugation, density gradient and centrifugation, and swim-up of overlaid semen [35,36,37]. Using a microfluidic device without laminar flow but with microchannel hydrodynamic constrain to isolate motile sperm and the sperm chromatin dispersion assay, which detects primarily single-strand DNA breaks, Quinn and colleagues [38] demonstrated significantly reduced DNA fragmentation index (DFI) in microfluidic isolated motile sperm (median: 0%; intraquartile ranges (IQR): 0–2.4) compared to motile sperm isolated with density-gradient centrifugation with swim-up (median—6%; intraquartile ranges (IQR): 3–11.5).

The second microfluidic method for motile sperm isolation involves multiple narrow channels and sperm inertia [39]. These microfluidic devices incorporate a radial array of hundreds of microchannels, with motile sperm swimming from the inlet to the outlets away from dead cells, debris, and seminal plasma—resulting in a highly motile population of sperm, again with reduced DNA damage compared to other conventional centrifugation-based semen-processing methods. Recently, these investigators have demonstrated the potential practical use of this design for human sperm isolation for clinical intracytoplasmic sperm injection [40].

Third, Wu and coworkers [41] have developed a microfluidic device that is able to generate an impeding flow field for isolating human motile sperm in a high-throughput manner. While a highly motile population of sperm is isolated in this device, the influence on sperm DNA integrity is unknown; yet, in theory, one would expect reduced processing-induced DNA fragmentation as demonstrated by the other microfluidic methods mentioned above. It is important to appreciate that these microfluidic devices do not directly improve sperm DNA integrity, but they do allow isolation of motile sperm—whereas raw samples have both motile (live) and non-motile (many times dead and DNA fragmented)—without processing-induced DNA damage. Finally, it is important to recognize that all of the above microfluidic devices and methods rely on spermatozoa motility for isolation. As mentioned earlier, testicular spermatozoa at the initial time of retrieval are predominantly non-motile; thus, other creative microfluidic methods or combinations of methods need to be considered in non-motile spermatozoa isolation from NOA testicular samples.

As mentioned above, microfluidics can circumvent centrifugation and deleterious influence on spermatozoa form and function experienced in conventional sperm processing. If one is using enzymatic processing of testicular samples to yield spermatozoa, then centrifugation can be part of the process. However, manual/mechanical processing does not necessarily entail centrifugation. An advantage of microfluidics isolation of spermatozoa from NOA testicular samples compared to other developing methods (magnetic-activated cell sorting (MACS) and fluorescence-activated cell sorting (FACS)—reviewed in Mangum et al., 2020 [42]) is the ability to isolate testicular spermatozoa without biochemical fluorescent or bead labeling of cells [43], which presents safety issues that are yet to be fully evaluated in gametes, fertilization, and offspring health [44]. Magnum and colleagues very nicely have provided a summary table of the advantages and disadvantages of these developing technologies. There are additional microfluidic approaches with potential for isolating non-motile testicular spermatozoa that have been proposed or proof-of-concept tested in animal models, such as combined microfluidics and dielectrophoresis cell sorting [45] and pinched flow fractionation [46,47]. Whether these microfluidic approaches and add-ons will be useful and/or beneficial in isolation of non-motile spermatozoa from human testicular samples of NOA men remains to be demonstrated.

## 4. Spiral Microfluidics, Inertial Separation, and Cell Size

As testicular resident spermatozoa are largely non-motile, the use of microfluidic laminar flow for isolation is not useful. Son and colleagues [48] demonstrated an ingenious and novel application of spiral microfluidics to effectively and efficiently separate non-motile spermatozoa (or beads of similar size) from non-motile cells of differing size. This spiral inertial microfluidic device yields separation of particles or cells based on size and shape. Spiral microchannel dimensions were calculated with specific consideration in relation to the cellular constituents of a single-cell suspension of a human testicular sample (spermatozoa, white blood cells (WBCs), and germ cells of a more immature state). This prototype spiral microfluidic device had a single inlet and multiple outlets to separate particles/cells at their equilibrium positions as they exit the device. Calculations were performed considering the differing cell sizes, various flow rates, and best conditions for cell focusing (microchannel height—50 μm, microchannel width—150 μm, space between microchannels—310 μm, initial radius—700 μm, and final radius—899 μm). The authors were able to demonstrate separation and isolation of spermatozoa from WBCs. More recently, Vasilescu and coworkers [49] used a similar spiral microchannel device produced by 3D printing to demonstrate rapid spermatozoa recovery from heterogeneous cell suspension of spermatozoa, WBCs, red blood cells, epithelial cells, and leukemic cancer cells. This study demonstrated rapid (5 min) separation of spermatozoa from other cell types and, very importantly, that this spiral microfluidic processing had no detrimental influence on spermatozoa viability, morphology, or DNA integrity. Collectively, these are exciting findings in the quest for future means of isolating immobile spermatozoa from testicular samples, yet there are some issues that remain uninvestigated and require testing. First, does spermatozoa shape/size asymmetry impact isolation efficiency? Second, while most testicular spermatozoa are non-motile, some testicular sperm can exhibit a form of motility termed “twitching”—how that might impact spiral microfluidic separation and focus isolation remains to be determined. This gives rise to a secondary issue of the need for determining the viability of spiral microfluidic testicular non-motile sperm post-isolation prior to use in ICSI. However, future combinations of spiral inertial microfluidic testicular spermatozoa isolation with a short culture period to induce maturation/some motility [20] or a non-terminal viability test, such as the hypo-osmotic swelling test of spermatozoa membrane integrity [50], may aid in addressing this issue.

## 5. Practical and Future Considerations of Using Microfluidics in Spermatozoa Isolation from NOA Testicular Samples

When initially considering the use of microfluidic applications with existing methods of gamete isolation, in vitro fertilization, embryo culture, gamete/embryo analysis, and/or cryopreservation, we need to first examine the practical shortcomings of the existing techniques, the potential benefits of incorporating microfluidics, and the potential hurdles that this incorporation of microfluidics may have in individual ART procedures. This leads to the practical question of why one might use microfluidics for non-motile spermatozoa isolation from retrieved NOA testicular samples. At a basic level, use of microfluidics would be justified if it does: (1) something we cannot do today; (2) something we do today, but is more efficient or provides a better sample; (3) something we do today, but is less expensive or requires less work, supplies, or personnel effort; (4) something we do today, as well as reduces intra-laboratory personnel and/or inter-laboratory variability; or (5) something we do today, but facilitates future automation and associated benefits [51]. While current methodologies of spermatozoa isolation from NOA testicular samples are manually burdensome and tedious, they do work on most occasions. Whether microfluidics will reduce the cases of “no spermatozoa found for ICSI”, increase efficiency, and/or produce a better sample in relation to fertilization rates, embryo development, and live-birth rates remains to be demonstrated. Use of microfluidics to isolate sperm from testicular samples will not become less expensive unless the personnel workload is significantly reduced. This could be the case in the future; however, it is important to recognize that most of the burdensome and tedious manual work in processing testicular samples is in producing a single-cell suspension, which is still needed for current microfluidic application to non-motile spermatozoa isolation from testicular samples. This brings up the potential hurdle of microfluidic application to non-motile spermatozoa isolation—specifically, the lack of microchannel functionality and/or clogging that can and will occur if input samples are not in a single-cell suspension. Notwithstanding the above discussion, the potential use of microfluidics in isolating non-motile spermatozoa from NOA testicular samples should continue to be investigated in rigorous and practical ways. Integration of multiple technologies—existing and of the future—will likely facilitate the use of microfluidics for improving success, reducing technical signatures and variation, and providing bridges over current limitations. Potential examples include combined spiral microfluidics [48,49] with subsequent short-term culture to assess viability/motility [20] and Raman spectroscopy to non-invasively interrogate sperm DNA integrity [52,53].
